# *Hsp90* and hepatobiliary transformation during sea lamprey metamorphosis

**DOI:** 10.1186/s12861-015-0097-2

**Published:** 2015-12-01

**Authors:** Yu-Wen Chung-Davidson, Chu-Yin Yeh, Ugo Bussy, Ke Li, Peter J. Davidson, Kaben G. Nanlohy, C. Titus Brown, Steven Whyard, Weiming Li

**Affiliations:** Departments of Fisheries and Wildlife, Michigan State University, 13 Natural Resources Building, 480 Wilson Road, East Lansing, MI 48824 USA; Physiology & College of Osteopathic Medicine, Michigan State University, East Lansing, MI 48824 USA; Computer Science & Engineering, Michigan State University, East Lansing, MI 48824 USA; Microbiology & Molecular Genetics, Michigan State University, East Lansing, MI 48824 USA; Department of Biological Sciences, University of Manitoba, Winnipeg, MB Canada

**Keywords:** Transcriptome, Geldanamycin, Bile acid, *cyp7a1*, Biliary atresia

## Abstract

**Background:**

Biliary atresia (BA) is a human infant disease with inflammatory fibrous obstructions in the bile ducts and is the most common cause for pediatric liver transplantation. In contrast, the sea lamprey undergoes developmental BA with transient cholestasis and fibrosis during metamorphosis, but emerges as a fecund adult. Therefore, sea lamprey liver metamorphosis may serve as an etiological model for human BA and provide pivotal information for hepatobiliary transformation and possible therapeutics.

**Results:**

We hypothesized that liver metamorphosis in sea lamprey is due to transcriptional reprogramming that dictates cellular remodeling during metamorphosis. We determined global gene expressions in liver at several metamorphic landmark stages by integrating mRNA-Seq and gene ontology analyses, and validated the results with real-time quantitative PCR, histological and immunohistochemical staining. These analyses revealed that gene expressions of protein folding chaperones, membrane transporters and extracellular matrices were altered and shifted during liver metamorphosis. HSP90, important in protein folding and invertebrate metamorphosis, was identified as a candidate key factor during liver metamorphosis in sea lamprey. Blocking HSP90 with geldanamycin facilitated liver metamorphosis and decreased the gene expressions of the rate limiting enzyme for cholesterol biosynthesis, HMGCoA reductase (*hmgcr*), and bile acid biosynthesis, *cyp7a1*. Injection of *hsp90* siRNA for 4 days altered gene expressions of *met*, *hmgcr*, *cyp27a1*, and *slc10a1*. Bile acid concentrations were increased while bile duct and gall bladder degeneration was facilitated and synchronized after *hsp90* siRNA injection.

**Conclusions:**

HSP90 appears to play crucial roles in hepatobiliary transformation during sea lamprey metamorphosis. Sea lamprey is a useful animal model to study postembryonic development and mechanisms for *hsp90*-induced hepatobiliary transformation.

**Electronic supplementary material:**

The online version of this article (doi:10.1186/s12861-015-0097-2) contains supplementary material, which is available to authorized users.

## Background

Metamorphosis represents a dramatic and large-scale morphological and functional change during post-embryonic development in free-living larvae of invertebrates and non-mammalian vertebrates including fishes, amphibians, and reptiles [1–6]. It has been extensively studied in insects and amphibians, and its developmental process is tightly controlled by hormones [5]. In general, metamorphosis in vertebrates is a single, uninterrupted larval-adult transition, whereas in insects and some other invertebrates, it proceeds through multiple larval and pupal moults, each repeated with a qualitatively similar hormonal interaction [5]. This distinction is exemplified for distinct pulses of ecdysone just preceding each larval and pupal moult in *Manduca sexta* and the single burst of triiodothyronine (T_3_) for metamorphosis of *Xenopus* larvae to the froglet stage [5].

In most chordates studied to date, the onset of metamorphosis is characterized by a peak of a thyroactive compound, activating the thyroid receptor that modifies the expression of target genes and leads to morphological remodeling characteristic of the larva-to-juvenile transition [4]. However, thyroid hormone did not seem to be the main factor controlling hind limb development in tadpoles [7] and metamorphosis in sea lamprey (*Petromyzon marinus* Linnaeus) [8–14]. In fact, there is a drop in circulatory thyroid hormone levels prior to metamorphosis, and treatment of thyroid hormones *per se* failed to induce metamorphosis in sea lamprey [8–14].

In invertebrates, HSP90 seems to be the main factor controlling metamorphosis. Blocking HSP90 function with geldanamycin triggers metamorphosis in protozoan Leishmania parasites [15] and in all major branches of metazoa including nematodes [16], molluscs [17] and sea urchin to tunicates [18, 19]. The sea lamprey, a jawless vertebrate, diverged from urochordates 550 million years ago [20–22]. The developmental control of sea lamprey metamorphosis may be an evolutionary intermediate between the HSP90-dependent invertebrate form and thyroid hormone-dependent vertebrate form [1]. Therefore, the sea lamprey presents a unique model to study the evolutionary transition of developmental control during metamorphosis.

The sea lamprey develops through distinct life stages [23, 24]. After hatching, larval sea lamprey live in burrows as benthic filter feeders. After seven metamorphic stages of dramatic change in external morphology and reorganization of internal organs [25], the emerging juveniles (JV) enter a parasitic phase during which they feed on blood and tissue fluid from host fish. After 1.5 to 2 years feeding in the ocean or large lakes, the adults cease feeding in the early spring and migrate into rivers to spawn and die [23, 24].

The hepatobiliary system undergoes the most dramatic changes during sea lamprey metamorphosis, compared to other organs such as the intestine and the kidney [26–28]. The cholangiocytes lining the extrahepatic bile duct and the gallbladder undergo apoptosis starting at the onset of metamorphosis (late larval stage; L), with the most dramatic morphological changes at metamorphic stage 2 (M2) and full degeneration at metamorphic stage 3 [26–28]. Occasionally one or two intrahepatic bile ducts persist into metamorphic stages 5 and 6, but usually disappear by stage 7 [27, 28]. The hepatocytes cease bile acid synthesis in the early metamorphic stages, undergo cyto-architectural reorganization, eventually resume bile acid synthesis at metamorphic stage 5 (M5) and proliferate to fill the space once occupied by the biliary system [27–30]. Despite thorough characterization of the cellular and organ-level morphological changes during sea lamprey metamorphosis, the developmental regulation of the hepatobiliary transformation is not fully understood.

We hypothesized that the hepatobiliary transformation during sea lamprey metamorphosis was due to transcriptional reprogramming that dictated cellular remodeling during metamorphosis, especially in landmark stages (L, M2, M5 and JV). We compared sea lamprey hepatobiliary transcriptomes at these landmark stages using mRNA-Seq and gene ontology (GO) analyses, and validated the sequencing results with real-time quantitative PCR (RTQ-PCR), histological and immunohistochemical staining, and antagonist and siRNA blocking experiments. Our results suggest that *hsp90* may be critical for the transformation of the hepatobiliary system during sea lamprey metamorphosis.

## Results

### Hepatobiliary transcriptome reprogramming during liver metamorphosis

**Fig. 1:**
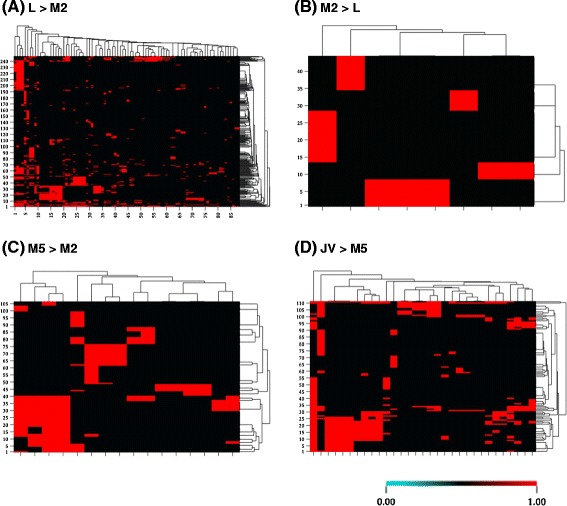
Gene ontology (GO) analyses of liver transcriptomes in metamorphic land mark stages. X-axis represents the GO categories and Y-axis represents gene clusters (listed in Additional file 1). Color scale represents the Log_2_ (transcript number in transcriptome 1/transcript number in transcriptome 2). Only genes with 2-fold changes (or greater) are shown. **a** Heat map showing GO categories at least 2-fold higher in larval (L) compared to metamorphic stage 2 (M2), *p* < 0.05, false discovery rate ≤ 0.1. **b** Heat map showing GO categories at least 2-fold higher in M2 compared to L, *p* < 0.05, false discovery rate ≤ 0.1. **c** Heat map showing GO categories at least 2-fold higher in metamorphic stage 5 (M5) compared to M2, *p* < 0.05, false discovery rate ≤ 0.1. **d** Heat map showing GO categories at least 2-fold higher in newly transformed juvenile (JV) compared to M5, *p* < 0.05, false discovery rate ≤ 0.1

We sequenced and compared the liver transcriptomes of L, M2, M5 and JV stages (Fig. 1). All sequencing reads were 75mers. From the L liver, 21,357,947 reads were sequenced, and 70.2 % of them passed the quality filter (14,985,824 reads). From the M2 liver, 19,272,978 reads were sequenced and 76.5 % of them passed the quality filter (14,747,950 reads). The M5 liver produced 22,479,660 reads, and 66.0 % of them passed the quality filter (14,834,568 reads). The JV liver produced 20,649,552 reads, and 70.2 % of them passed the quality filter (14,490,540 reads). These sequences were assembled and aligned to a total of 3246 genes, and these genes were clustered into 5297 GO categories. The GO analyses compared differential expression (≥2-fold difference) between the transcriptomes of different life stages.

**Fig. 2:**
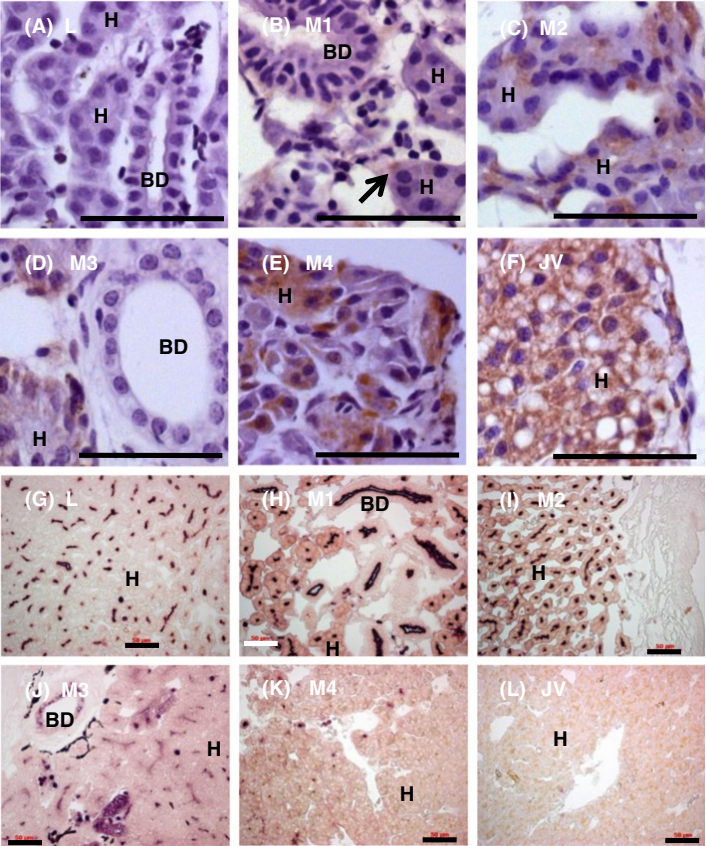
Hepatocyte proliferation and cholangiocyte apoptosis during liver metamorphosis. **a**–**f**: Mitosis marker phospho-histone 3 immunostaining (brown) in liver section at various metamorphic stages. Paraffin sections (4 μm) were stained with DAB and counterstained with hematoxylin (blue/purple) nuclear stain. Note that staining is localized only in hepatocytes. **g**–**l**: Apoptosis marker alkaline phosphatase staining (dark purple) in liver section at various metamorphic stages. Paraffin sections (4 μm) were stained with alkaline phosphatase (AP) substrate NBT/BCIP and counterstained with nuclear Fast Red (pink/red stain). In larval (L) and metamorphic stages 1 (M1) and 2 (M2), AP activity was located in canaliculi between hepatocytes and the lumen lining of the cholangiocytes. In metamorphic stage 3 (M3), AP activity was located in canaliculi between hepatocytes, blood cells and whole cholangiocytes. In metamorphic stage 4 (M4), only a few blood cells contain AP activity. In newly transformed juvenile (JV), no AP activity was detected. Scale bar: 50 μm. BD: bile duct; H: hepatocyte

Comparison of L and M2 liver transcriptomes revealed that 88 GO categories and 248 genes were expressed at least 2-fold higher in L, whereas 8 GO categories and 44 genes were expressed at least 2-fold higher in M2. Gene ontology categories related to developmental and metabolic processes and cell division (mitosis) were more active in L compared to M2 (legends for x-axis, Fig. 1a). Signaling pathways (phosphoinositide-mediated and second-messenger-mediated signaling) and cytokine production were also higher in L compared to M2 (Fig. 1a). On the contrary, genes involved in protein folding and transmembrane transport were up-regulated at M2 compared to L (Fig. 1b). At M2, the response to extracellular and external stimuli, and nutrient levels also increased compared to L (Fig. 1b). Phospho-histone 3 (p-H3, a mitotic marker) immunostaining confirmed that cell division was occurring in hepatocytes (Fig. 2, a–f). On the other hand, cholangiocytes were apoptotic and degenerated, as indicated by alkaline phosphatase staining (Fig. 2, g–l).

Comparison of M2 and M5 liver transcriptomes revealed that 16 GO categories and 106 genes were expressed at least 2-fold higher at M5. Tissue development, lipid metabolic processes, cell division, and phosphoinositide-mediated signaling pathways were higher in M5 compared to M2 (legends for x-axis, Fig. 1c). Phospho-histone 3 immunostaining confirmed that cell division was occurring in hepatocytes (Fig. 2, a–f). Most bile ducts disappeared by M4 (Fig. 2).

Comparison of M5 and JV liver transcriptomes revealed that 31 GO categories and 111 genes were expressed at least 2-fold higher in JV, mostly related to increased small molecule and organic acid (including bile acid) metabolism in the liver (legends for x-axis, Fig. 1d). Cell proliferation was reduced at JV (p-H3 immunostaining, Fig. 2f), and apoptosis was rarely detected (alkaline phosphatase staining, Fig. 2l).

### Differential transporter gene expression during liver metamorphosis

**Fig. 3:**
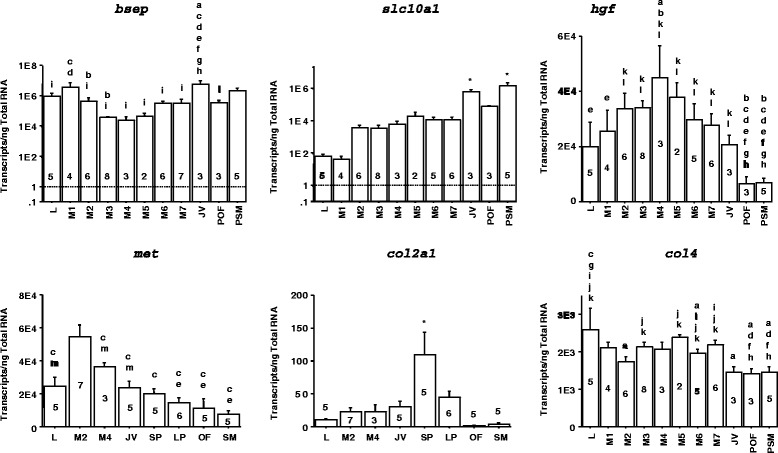
Differential gene expressions in sea lamprey liver at various metamorphic and life stages. Selected genes: bile salt export pump (*bsep*), sodium/bile acid co-transporter (*slc10a1*), hepatocyte growth factor (*hgf*), HGF receptor *met*, collagen *col2a1*, and collagen *col4*. a: significantly different from larval (L) group; b: significantly different from metamorphic stage 1 (M1); c: significantly different from M2; d: significantly different from M3; e: significantly different from M4; f: significantly different from M5; g: significantly different from M6; h: significantly different from M7; i: significantly different from newly transformed juvenile (JV); j: significantly different from pre-ovulatory female (POF); k: significantly different from pre-spermiating male (PSM) group; l: significantly different from ovulatory female (OF); m: significantly different from spermiating male (SM); *: significantly different from all other groups (ANOVA test followed by post hoc test, *p* < 0.05). A: adult; LP: large parasite; SP: small parasite. Number on each bar represents the number (n) of lamprey in each group

The transporters expressed at landmark metamorphic stages varied dramatically (Table 1), and provided a snapshot of the essential factors required at each metamorphic stage. It appeared that liver transporter gene expressions shifted during sea lamprey metamorphosis. At L, transporters for phospholipids or fatty acids, Ca^2+^, amino acids, peptides or oligopeptides were more prominent than M2. At M2, metabolism and bile acid transport were down-regulated, whereas the gene expressions of transporters for wound healing molecules (thiamin and vitamin C) and cytoprotective agents such as taurine were dramatically increased. Bile salt export pump (*bsep*) gene expression increased at later metamorphic stages and JV (Fig. 3). Expression of bile acid co-transporter *slc10a1* was increased in JV, but not at M2 (Table 1, Fig. 3).

**Table 1:** Transporter gene expression at various metamorphic stages

Transporters	Transported molecules	L	M2	M5	JV
*abca1*	Cholesterol	+	+	+	+
*abcb4*	Phosphotidylcholine (hepatocyte to bile)		+	++	
*abcb6*	Unknown (Half transporter, mitochondrial)		++	+	
*abcb9*	Unknown (Half transporter, lysosomal)			+	++
*abcc5*	Cyclic nucleotide			+	++
*abcd2*	Fatty acid, fatty acyl-CoA (Half transporter, peroxisomal)				++
*abcg2*	Xenobiotic		+	++	
*atp1a2*	Na^+^/K^+^		+	++	++
*atp1b3*	Na^+^/K^+^		+	++	+++
*atp2a1*	Ca^2+^ (cytosol to ER)	+	++		
*atp2a2*	Ca^2+^(cytosol to ER)	++	+		
*atp2b1*	Ca^2+^ (Plasma membrane)			+	++
*atp5j2*	H^+^ (Mitochondrial)		+	++	
*atp5o*	H^+^ (Mitochondrial)	+	++		++
*atp6v1f*	H^+^ (Mitochondrial)	+	++		
*atp6v1h*	H^+^ (Mitochondrial)	+	++		
*atp8a1*	Aminophospholipid (phosphotidyl serine)		++	+	
*atp10d*	Phospholipid	++	+	++	
*slc1a1*	Glu		+	++	
*slc2a9*	Glucose		++	+	
*slc4a4*	Na^+^, bicarbonate	++	+		
*slc4a11*	Na^+^, borate	++	+	++	++
*slc5a6*	Na^+^, vitamin (pentothenate, biotin, lipoate)	+	++	+	+++
*slc5a7*	Na^+^, Cl^-^/Choline	+	++	+	
*slc6a2*	Na^+^, NE			+	++
*slc6a6*	Na^+^/taurine, β-Ala	+	++	+	
*slc6a8*	Creatine			+	++
*slc6a12*	GABA, betaine	++	+	++	+++
*slc7a1*	Cationic amino acid				++
*slc7a5*	Cationic amino acid	++	+	++	
*slc7a6*	Na^+^/cationic amino acid	++	+	++	
*slc7a8*	Neutral amino acid	+	++	+	
*slc7a11*	Cationic amino acid	++	+	++	++
*slc9a7*	Na^+^/H^+^	+	++	+	+++
*slc10a1*	Na^+^, bile acids (basal)	++	++	+++	++++
*slc12a2*	Na^+^/K^+^/Cl^-^	++	+		
*slc12a6*	K^+^, Cl^-^	++	+	++	+++
*slc15a1*	H^+^/peptide	++	+	++	
*slc15a2*	H^+^/peptide	++	+	++	
*slc16a4*	H^+^/monocarbohydrates	+	++	++	+++
*slc18a2*	Monoamine (vesicular)	++	+		
*slc19a2*	Thiamine	+	++		
*slc20a1*	Na^+^, phosphate	+	++		
*slc20a2*	Na^+^, phosphate	+	++	+++	+++
*slc22a3*	Organic cations	+	++	+	++
*slc22a18*	Organic cations	+	++		
*slc23a1*	Vitamin C	+	++	+	
*slc23a2*	Vitamin C	+	++	+	
*slc25a1*	Citrate-H^+^, malate (Mitochondrial)	+	++	++	+++
*slc25a3*	Phosphate (cytosol to mitochondrial)		+	++	
*slc25a5*	ADP (mitochondrial to cytosol)		+	++	
*slc25a13*	H^+^/Asp, Glu (mitochondrial)		+	++	
*slc25a26*	Metabolites, cofactors (mitochondrial)				++
*slc25a36*	Unknown				++
*slc26a5*	Cl^-^, bicarbonate	+	++		
*slc27a6*	Fatty acid	++	+		
*slc29a1*	Nucleoside				++
*slc30a1*	Zn^2+^				++
*slc30a2*	Zn^2+^	++++	+++	++	++
*slc35a3*	UDP-GlcNac	++	+		
*slc35b1*	UDP-galactose	+	++	+	
*slc35b2*	3′-phospho-5′-phosphosulfate	+	++		
*slc35b4*	UDP-xylose, UDP-GlcNAc	+	++		
*slc37a4*	Glucose-6-phosphate			+	++

*L* larva, *M2* metamorphic stage 2, *M5* metamorphic stage 5, *JV* newly transformed juvenile, *+* expressed with at least 2-fold difference between paired transcriptomes

### HGF and MET promote hepatocyte regeneration during liver metamorphosis

Real time quantitative PCR analyses of liver gene expressions throughout various life stages revealed that hepatocyte growth factor (*hgf*), a hepatocyte proliferating agent, increased at M4, whereas its receptor *met* peaked at M2 (Fig. 3). These results demonstrated that *hgf* gene expression peaked at the metamorphic stage with heightened hepatocyte proliferation (M4).

### Alteration of Extracellular Matrix (ECM) composition during liver metamorphosis

**Fig. 4:**
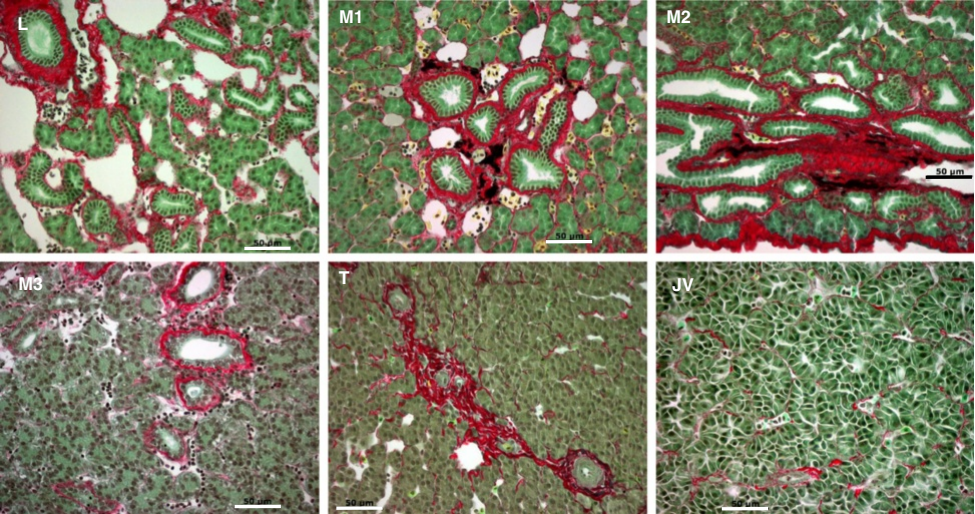
Collagen fiber reorganization during liver metamorphosis in sea lamprey. Transient fibrosis (thickened red collagen deposit) was observed around proliferating bile ducts during liver metamorphosis. Paraffin sections were stained with Picro Sirius Red (Gladstone). Scale bar: 50 μm. L: larval stage, M1-3: metamorphic stages 1 –3, T: newly transformed juvenile, JV: juvenile

The extracellular matrix (ECM) composition changed during liver metamorphosis (Table 2). Collagen gene *col3a1* expression peaked at L stage whereas *col2a1* expression peaked at JV stage (Table 2). Collagen genes *col1a2*, *col4a4*, and *col5a2*, as well as laminin *lamc1* were up-regulated from M2 to JV stages, whereas *col12a1* was down-regulated during metamorphosis (Table 2). Collagen genes *col4a6*, *col5a1*, and *col6a2* were down-regulated only at M2 while *col4b6* was down-regulated at L and M2 (Table 2). The general trend was a transition from *col1a1* to *col1a2* for Type I collagen, a switch from Type III to Type II collagens, an induction from a single subtype to multiple subtypes for Type IV and Type V collagens, and the increase in the non-collagenous constituent of the basement membrane *lamc1*. Type VI and Type XII collages were generally down-regulated during metamorphosis. Real-time quantitative PCR confirmed that *col2a1* and *col4* gene expressions were consistent with the transcriptome results (Fig. 3). Picro Sirius Red staining for collagen fibers revealed a transient fibrosis during liver metamorphosis, which later cleared (Fig. 4).

**Table 2:** Extracellular matrix gene expression at various metamorphic stages

Symbol	Full name	L	M2	M5	JV
*col1a1*	Collagen, Type I, Alpha-1	+		+	
*col1a2*	Collagen, Type I, Alpha-2		+	++	+
*col2a1*	Collagen, Type II, Alpha-1				+
*col3a1*	Collagen, Type III, Alpha 1	+			
*col4a4*	Collagen, Type IV, Alpha 4		++	+	+
*col4a6*	Collagen, Type IV, Alpha 6	+		+	+
*col4b6*	Collagen, Type IV, Beta 6			+	+
*col5a1*	Collagen, Type V, Alpha 1	+		++	++
*col5a2*	Collagen, Type V, Alpha 2		+	++	++
*col6a2*	Collagen, Type VI, Alpha 2	++		++	+
*col12a1*	Collagen, Type XII, Alpha 1	+			++
*lamc1*	Laminin, Gamma 1		+	+	+
*muc5ac*	Mucin 5 AC, Oligomeric Mucus/Gel-Forming	+++	++		+

*L* larva, *M2* metamorphic stage 2, *M5* metamorphic stage 5, *JV* newly transformed juvenile, + expressed with at least 2-fold difference between paired transcriptomes

### Hsp90 gene expression altered at landmark developmental event

**Fig. 5:**
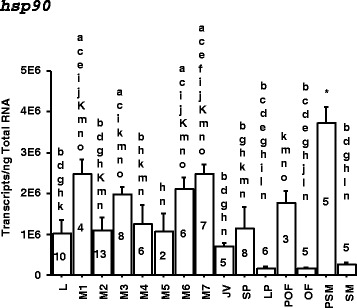
*Hsp90* gene expression during various metamorphic and life stages in the liver of sea lamprey. Increased *hsp90* mRNA expression coincided with the onset (M1), gall bladder disappearance (M3), and final stages (M6-M7) of metamorphosis. Sharp declines of *hsp90* mRNA concentrations also demarcated the heightened biliary degeneration stage (M2) and the transition in life stages (M → T, SP → LP, POF → OF, and PSM → SM). a: significantly different from larval (L) group; b: significantly different from metamorphic stage 1 (M1); c: significantly different from M2; d: significantly different from M3; e: significantly different from M4; f: significantly different from M5; g: significantly different from M6; h: significantly different from M7; i: significantly different from newly transformed juvenile (JV); j: significantly different from small parasite (SP); k: significantly different from large parasite (LP); l: significantly different from pre-ovulatory female (POF); m: significantly different from ovulatory female (OF); n: significantly different from pre-spermiating male (PSM); o: significantly different from spermiating male (SM); *: significantly different from all other groups (ANOVA test followed by post hoc test, *p* < 0.05). Number on each bar represents the number (n) of lamprey in each group

Transcriptome analyses revealed that genes involved in protein folding were up-regulated at M2 when the most dramatic metamorphic events occurred. *Hsp90* was identified as a candidate key factor during liver metamorphosis because of its involvement in protein folding [31], metamorphosis in invertebrates [15, 18], and human biliary atresia [32]. Increased *hsp90* mRNA expression coincided with the onset (M1), gall bladder disappearance (M3), and final stages (M6-M7) of metamorphosis (Fig. 5). Sharp declines of *hsp90* mRNA concentrations also demarcated the heightened biliary degeneration stage (M2) and transitions in metamorphic and life stages, for example: metamorphic stage 7 (M7) to newly-transformed juvenile (T), small parasite (SP) to large parasite (LP), pre-ovulatory female (POF) to ovulatory female (OF), and pre-spermiating male (PSM) to spermiating male (SM) (Fig. 5).

**Fig. 6:**
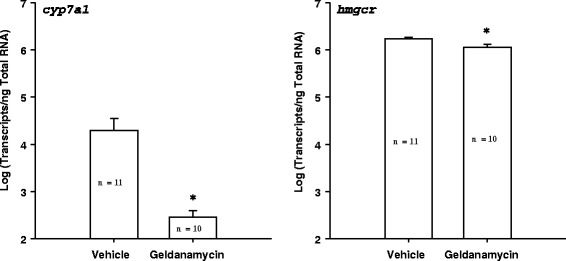
Geldanamycin (HSP90 inhibitor) decreased *cyp7a1*and HMGCoA reductase (*hmgcr)* transcriptions in sea lamprey liver. *: significantly different from the control group (t-test and F-test, *p* < 0.05)

**Fig. 7:**
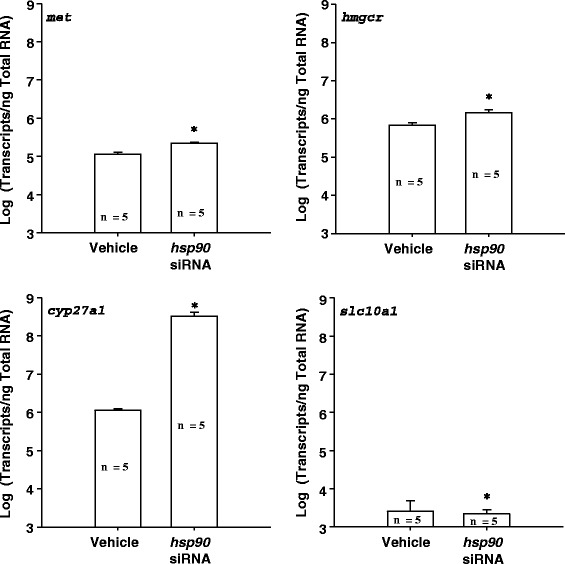
*Hsp90* siRNA treatment altered gene expressions in sea lamprey liver during metamorphosis. Hsp90 siRNA treatment increased *met*, HMGCoA reductase (*hmgcr*), and *cyp27a1*, but decreased *slc10a1* gene expressions (t-test and F-test, *p* < 0.05)

**Fig. 8:**
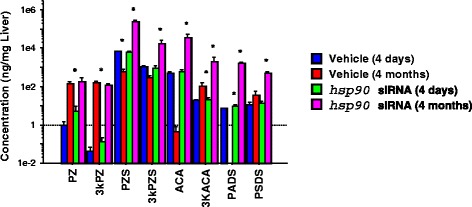
*Hsp90* siRNA treatment increased bile acid concentrations in sea lamprey liver during metamorphosis. Hsp90 siRNA treatment increased bile acids 3 keto-petromyzonol sulfate (3kPZS), petromyzonol sulfate (PZS), allocholic acid (ACA), 3-keto allocholic acid (3kACA), petromyzonol (PZ), 3 keto-petromyzonol (3kPZ), petromyzonamine disulfate (PADS), and petromyzonsterol disulfate (PSDS) (ANOVA test for repeated measurement, followed by post hoc tests, *p < 0.05)

**Fig. 9:**
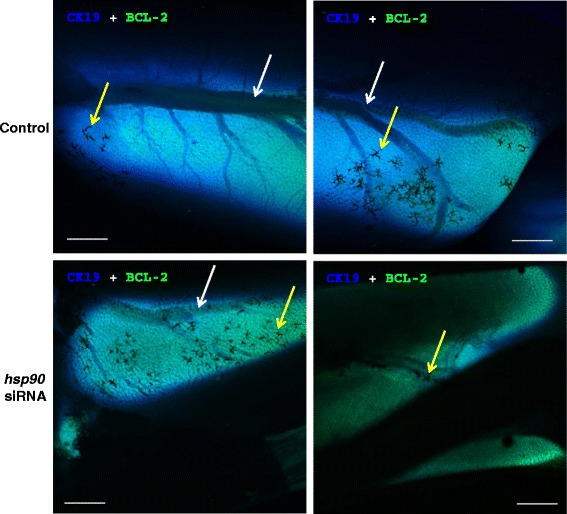
*Hsp90* siRNA treatment decreased CK19 and BCL2 immunofluorescence in sea lamprey liver during metamorphosis. Confocal images showing the biliary tree in sea lamprey liver (whole organ processed with a modified CLARITY method [73]) during metamorphosis. Left panels are taken from the anterior, and the right panels from the posterior end. Images of liver cell surface marker cytokeratin 19 (1:100 mouse-anti-CK19, Thermo Fisher; stained with 2 μg/ml Alexa Fluor 350-goat-anti-mouse IgG, Life Technologies; blue fluorescence) and anti-apoptotic marker BCL2 (1:100 rabbit-anti-Bcl2, Santa Cruz; stained with 2 μg/ml Alexa Fluor 488-donkey-anti-rabbit IgG, Life Technologies; green fluorescence) are superimposed. White arrows indicate the biliary tree. Yellow arrows indicate the dead cells and surrounding canaliculi and ductules in the liver. Note that the biliary tree was mostly degenerated in *hsp90* siRNA-treated liver. Scale bar: 500 μm

The HSP90 blocker geldanamycin facilitated and *hsp90* siRNA synchronized gall bladder degeneration during liver metamorphosis in sea lamprey (Table 3; Additional file 1). The vehicle control group showed a wide range of gall bladder sizes (1.775 ± 0.300 mm, mean ± S.E.M) whereas the gall bladder of the *hsp90* siRNA-treated group was more homogeneous in sizes (1.833 ± 0.102 mm, mean ± S.E.M.) (F-test, dF = 7, *p* < 0.05). Geldanamycin treatment decreased the rate-limiting enzymes in the cholesterol (*hmgcr*) and classical bile acid (*cyp7a1*) synthetic pathways, but did not alter the expression of *hsp90* (Fig. 6). In addition, *hsp90* siRNA treatment altered the gene expressions of *met* (receptor for hepatocyte growth factor), *hmgcr*, *slc10a1* and the rate-limiting enzyme for alternative bile acid synthetic pathway, *cyp27a1* (Fig. 7), but had no effect on *cyp7a1*. Bile acid concentrations were increased 4 days and 4 months after *hsp90* siRNA injection (Fig. 8). Bile duct degeneration was facilitated at 4 months after *hsp90* siRNA injection (Fig. 9).

**Table 3:** HSP90 antagonist geldanamycin facilitates metamorphosis in sea lamprey

Metamorphic stage	Frequency in the control group	Frequency in the 5 μM geldanamycin-treated group
1	2	0
2	6	0
3	3	7
4	0	3
5	0	0
6	0	0
7	0	0
Fully transformed juvenile	0	0

χ^2^ = 12.581, DF = 3, *p* = 0.0056

## Discussion

We hypothesized that transcriptional reprogramming in landmark stages (L, M2, M5 and JV) drove hepatobiliary transformation during sea lamprey metamorphosis. Our results showed that hepatobiliary transcriptome reprogramming occurred at landmark stages during metamorphosis. The transporter genes and the ECM gene composition were altered during liver metamorphosis, and the histological and morphological changes during liver metamorphosis also paralleled the transcriptome reprogramming. These results are similar to the remodeling through “gene switching” observed in *Xenopus laevis* liver metamorphosis [33]. The changes in liver ECM composition also occurred during the transition from liver fibrosis and steatohepatitis to hepatocellular carcinoma [34].

Type IIA *col2a1* mRNA was the major form of transcript found in most nonchondrogenic tissues of early human embryos [35]. Similar to observations in the mouse, expression of *col2a1* transcripts in human embryos was generally transient and was found in epithelial structures that participated in epithelial-mesenchymal interactions [35]. Expression of *col2a1* mRNA was associated with major tissue remodeling such as hepatic cord development in the liver [35]. A previous morphological study showed that the onset of bile duct degeneration was demarcated by the folding and pleating of the basement membrane [27]. It is likely that the disintegration and reorganization of the basement membrane was associated with the early events of liver metamorphosis. Indeed, the breakdown of basement membranes was an important step in the controlled rearrangement of cells during metamorphosis [36]. Our observation of transient fibrosis during liver metamorphosis is consistent with these prior demonstrations that reorganization of collagen fibers plays a significant role in liver metamorphosis.

The cholangiocytes degenerated during liver metamorphosis in the sea lamprey, similar to those from analyses of Japanese lamprey (*Lethenteron reissneri*) metamorphosis, which showed that the apoptotic signal was initiated in the region around the cystic duct, and the smaller peripheral ducts degenerate more rapidly than the large central ducts [37]. Our results are also consistent with previous findings that membrane enzymes such as alkaline phosphatase relocated from apical to lateral membranes during lamprey BA [38].

Apoptosis of cholangiocytes in liver allograft rejection was attributed to down-regulation of the apoptosis-inhibitory BCL-2 protein [27]. Our transcriptome data showed that the gene expression of *bcl-2* was the highest at L stage compared to M2 and M5 (no difference between M2 and M5) and the lowest at JV stage. Between M2 and M5, sea lamprey transitions from the ductal to aductal stage, and cholangiocytes undergo apoptosis and degenerate. At JV stage, the liver of the transformed animal is totally aductal without any cholangiocytes. It is interesting that BCL-2 protein is absent in the early stage of human intrahepatic biliary system development, but present in the late stage [38], and apoptosis can be induced by the purine-scaffold HSP90 inhibitor PU-H71 via down-regulation of Bcl-2 [39]. In addition, inhibition of the molecular chaperone function of HSP90 by geldanamycin induces endoplasmic reticulum (ER) stress-mediated apoptosis in different cell lines [40–42]. It would seem that at L stage, low *hsp90* and high *bcl-2* expressions might protect against apoptosis.

The hepatocytes underwent mitosis during liver metamorphosis, indicated by some nuclear and mostly cytoplasmic staining of p-H3, a mitotic marker. H3 phosphorylation has been shown to initiate at early prophase, and spread throughout the chromosomes at late prophase. At metaphase, most of the p-H3 aggregates at the ends of the condensed chromosomes at the equatorial plate. During anaphase and telophase, p-H3 is usually detached from chromosomes and found in the cytoplasm [43]. Our results indicated that most of the hepatocytes with p-H3 staining were at anaphase or telophase. Interestingly, histones could induce profound changes in the autophosphorylation of HSP90 [44], and the direct interaction of HSP90 and histones induced condensation of the chromatin structure [45] and decreased overall transcription activity [46]. HSP90 is evolutionarily conserved and essential for the maturation, activation and stability of “client” proteins that regulate proliferation, cell cycle and survival [31, 47]. Therefore, HSP90 could contribute to the activity of H3 phosphorylation and cell proliferation.

HGF has been shown to have potent effects on hepatic growth and differentiation [48–52]. It functioned as a complete mitogen for hepatocytes [53] and produced an anti-apoptotic effect [54, 55]. The MET signaling cascade is also a major player in liver development and differentiation [53–55]. It is possible that HGF and MET facilitated hepatocyte regeneration during sea lamprey metamorphosis. Interestingly, MET and its downstream effectors were also HSP90 clients [56, 57]. HGF and MET were both present in intrahepatic biliary system (IBS) cells in the early stage of human IBS development [38]. Inhibition of HSP90 increased *hgf* gene expression [58], and HSP90 inhibitor exhibited antineoplastic and antiangiogenic activity via MET [59]. It appears that *hsp90* is tightly linked to the activity of *met* during liver metamorphosis in sea lamprey.

Gene expression of *hsp90* decreased during metamorphosis but dramatically increased after metamorphosis in sea lamprey liver. This is consistent with the finding that blocking HSP90 function with the HSP90 inhibitor geldanamycin triggered metamorphosis in tunicates [19]. The transition from larval to adult stages in marine invertebrates was tightly regulated by an environmentally-sensitive competent period that delayed metamorphosis until the proper cues were detected by the larvae [60–62]. In sea urchins and ascidians, HSP90 inhibitors triggered metamorphosis when applied during the competent period [15]. HSP90 and its substrates were also involved in life-cycle transitions of Leishmania parasites [16] and the metamorphosis of species spanning all major branches of metazoan phylogeny, from insects and nematodes [18], to echinoderms [60], ascidians [17] and mollusks [63]. Even in the amphibian, other members of the *hsp* family such as *hsp30s* played an important role in the transformation of the liver of *Rana catesbeiana* during metamorphosis from an ammonotelic larva to a ureotelic adult [64]. Interestingly, we found that HSP90 blocker geldanamycin facilitated and *hsp90* siRNA treatment synchronized gall bladder degeneration during liver metamorphosis in the sea lamprey. Coincidentally, proteomic analyses comparing BA and non-BA neonatal cholestasis patients showed that HSP90 was the most significant biomarker that was down-regulated in BA patients [32].

Furthermore, we found that the expression of cholesterol and bile acid synthetic genes such as *hmgcr*, *cyp7a1*, and *cyp27a1*, and bile acid concentrations were altered after geldanamycin or *hsp90* siRNA injection, even up to 4 months. These results are consistent with reports that HSP90 was involved in cholesterol homeostasis [65, 66]. Lipid content was an important determining factor of lamprey metamorphosis [67], and lipid and bile acid biosynthesis and metabolism changed dramatically during sea lamprey metamorphosis [29, 68]. These results suggest that HSP90 was involved in many aspects of hepatobiliary transformation. However, geldanamycin or *hsp90* siRNA did not affect *hsp90* mRNA concentration *per se* at the time points examined. Further time course analyses are required to establish the actual relations between *hsp90* and all genes affected.

## Conclusions

During sea lamprey liver metamorphosis, the ECM may be associated with the reorganization of the liver architecture. At the early stage of metamorphosis, the metabolism of hepatocytes was down-regulated. Extracellular and external stimuli, nutrient levels, and phosphoinositide-mediated signaling pathways strongly affected the process of liver metamorphosis. Major shifts in transporter expressions occurred throughout liver metamorphosis, whereas hepatocytes proliferated at later stages. Once the process of biliary atresia was completed, the metabolism in hepatocytes resumed. *Hsp90* was involved in many aspects of hepatobiliary transformation including hepatocyte regeneration, biliary degeneration and changes in bile acid synthesis. We conclude that the sea lamprey is a useful animal model to study postembryonic development and mechanisms for *hsp90*-induced hepatobiliary transformation.

## Methods

### Collection and maintenance of animals

All animals received humane care according to the criteria outlined in the “Guide for the Care and Use of Laboratory Animals” prepared by the National Academy of Sciences and published by the National Institutes of Health (NIH publication 86-23 revised 1985) of the United States. Animal handling procedures were approved by the Institutional Animal Care and Use Committee at Michigan State University (MSU).

Larval lampreys, collected by the staff of U.S. Geological Survey Lake Huron Biological Station or by the survey crew of U.S. Fish and Wildlife Service Ludington Biological Station (Ludington, MI, USA), were kept in plastic tanks (98 × 54 × 48 cm^3^, length × width × height) with flow-through water. The bottom of the tanks were filled with 13 cm of fine sand, which can pass through No. 18 (1 mm), 3ϕ (125 μm) or 4ϕ (62.5 μm) U.S. standard sieve. Water tanks were aerated with stone air breakers to keep dissolved oxygen levels in the water near saturation. The water flow was kept at 10 L/min and the water temperature at 16 ± 1 °C. Larvae were fed with dried brewer’s yeast (60 g/100 larvae, suspended in 100 ml water before use) once per week. Larvae that had started metamorphic processes were checked every other week. Liver samples were collected from various metamorphic stages according to the appearance of eyes, the structure of oral aperture, the degree of development of the tongue-like piston, the shape and degree of cornification of the teeth, and the coloration of the body [25]. Due to the difficulty of obtaining metamorphic liver samples, sample sizes varied among experiments.

### GO analyses

Transcriptome expression data for sea lamprey liver at various metamorphic stages were obtained using an Illumina Genome Analyzer II (Illumina, Inc., San Diego, CA, USA) and mRNA-Seq protocol (75-mers, mRNA-Seq 8 sample Prep Kit, Illumina) at the Genomics Technology Support Facility in MSU. Two-way BLASTX between lamprey transcriptome ESTs and mouse protein database was performed to obtain putative orthologues. GO categories were assigned to the corresponding ESTs according to NCBI/Entrez databases. Bowtie software was used to quantify the number of reads [69]. The raw counts were normalized using quantile normalization. Normalized profiles were pairwise-compared using GoMiner software [70]. Heat maps were generated using CIMminer software [71].

### Histology and immunohistochemistry

Histological samples were processed in the Investigative Histopathology Laboratory at MSU. Immunostaining for p-H3 followed the methods described previously [72]. Negative controls (deprived of the primary antibody) were performed simultaneously in every immunostaining experiment. The antibody concentration used for p-H3 was 1:1000 (Sigma; St. Louis, MO, USA). Briefly, paraffin sections (4 μm) were deparaffinized with xylene and rehydrated through an ethanol series (100 to 10 %) and rinsed in Tris buffered saline (TBS: 50 mM Tris, 150 mM NaCl, pH 7.2; 5 min each). All reagents were diluted with TBS/0.05 % Triton-X100 according to the manufacturer’s instructions unless mentioned otherwise. Sections were incubated in the mixture of primary antibody for p-H3 (1:1000, Sigma) and normal goat serum (Vector Laboratories; Burlingame, CA, USA) over night at 4 °C. Sections were then incubated in biotinylated secondary antibody (goat-anti-rabbit IgG, Vector), followed by ABC solution (Vector), and stained with 3,3′-diaminobenzidine (Vector).

Alkaline phosphatase staining followed the methods described previously [72]. Briefly, paraffin sections were deparaffinized and rehydrated as described above, incubated with nitroblue tetrazolium chloride and 5-bromo-4-chloro-3 indolyl phosphate substrate (Roche Applied Science, Indianapolis, IN, USA) for 3 h, and counterstained with Nuclear Fast Red (Vector).

### Hsp90 antagonist and siRNA experiments

#### Hsp90 antagonist experiment

Thirty-three sea lamprey larvae at metamorphic stage 2 (M2) were treated with vehicle (0.05 % dimethyl sulfoxide, Sigma; interperitoneal (i.p.) injection) or 5 μM geldanamycin (Tocris Bioscience, Park Ellisville, MO, USA; i.p. injection, 50 μl/ml body volume). Animals were euthanized with 0.02 % MS222 (Sigma) after 1 week. Liver were snap-frozen in liquid nitrogen and stored at −80 °C before processing for real-time quantitative PCR.

### siRNA experiment I

Twenty sea lamprey larvae at metamorphic stage 2 (M2) were treated with vehicle (3.3 % lipofectamine, Life Technologies, Grand Island, NY, USA; i.p. injection) or 53.67 μg/ml *hsp90* siRNA (Stealth RNAi duplex with sense sequence: 5′GCAGCAAAGUGGCGUAUUA3′, and antisense sequence: 5′UAAUACGCCACUUUGCUGC3′, Life Technologies; i.p. injection, 50 μl/g body weight). Animals were euthanized with 0.02 % MS222 after 4 days. Liver were snap-frozen in liquid nitrogen and stored at −80 °C before processing for real-time quantitative PCR and LC-MS/MS.

### siRNA experiment II

Twenty sea lamprey larvae at metamorphic stage 2 (M2) were injected with vehicle or *hsp90* siRNA as in siRNA Experiment I every other week (4 injections total). Animals were euthanized with 0.02 % MS222 after 4 months. Half of the liver samples were snap-frozen in liquid nitrogen and stored at −80 °C before processing for LC-MS/MS. The other half were fixed in hydrogel and processed for CLARITY, immunofluorescent staining and confocal microscopy according to Chung-Davidson et al. [73].

### LC-MS/MS analyses of bile acids

Bile acid analyses followed the method developed by Li et al. [74] with minor modification. The analysis method was validated and exceeds the minimum standards recommended in the Food and Drug Administration guidance. Briefly, 1 ml 75 % ethanol and 10 ng internal standard ([^2^H_5_]3keto-petromyzonol sulfate; Bridge Organic Inc., Vicksburg, MI, USA) was added to the whole liver (24.9 ± 1.7 mg). Liver tissues were homogenized and incubated in a shaker with 70 rpm at room temperature overnight. The homogenized tissues were then centrifuged at 13,000 × g for 10 min. The supernatant was transferred to a new tube, freeze-dried overnight, and stored at −20 °C until analyses. Samples were reconstituted in 1 mL of methanol:water (1:1) and placed in an autosampler for LC-MS/MS analysis.

### Real-Time Quantitative PCR (RTQ-PCR)

RTQ-PCR was performed using the TaqMan MGB or SYBR Green system (Life Technologies) as described previously [29]. Gene sequences were obtained from the sea lamprey genome as described previously [75]. Synthetic oligos were used as standards and run simultaneously on the sample plate. The sequences for standards, primers and TaqMan MGB probe for each mRNA are listed below. Briefly, total RNA was extracted using TRIzol Reagent (Life Technologies), and treated with the TURBO DNA-free kit (Life Technologies). RNA samples were then reverse-transcribed into cDNA using M-MLV reverse transcriptase (Life Technologies) and random hexamers (Promega Corp.; Madison, WI, USA). Each RTQ-PCR reaction consisted of 2 μl (5 ng/ μl) cDNA, 8 μl TaqMan or SYBR Green Universal PCR master mix, 900 nM each forward and reverse primers, and 250 nM TaqMan MGB probe (for TaqMan MGB system only). Amplification plots were analyzed on an ABI 7900 real-time PCR thermal cycler (Life Technologies). 40S and 60S ribosomal RNAs were used as internal standards and were confirmed not to change in expression levels among treatment groups.

For each gene, the following information is shown: gene name and synthetic oligo used as the standard for RTQ-PCR. 5′ and 3′ primer sequences are underlined. (Note: the 3′ primer is complementary to the sequence shown). TaqMan MGB probes are shown in uppercase. **40S ribosomal protein**: 5′acctacgcaggaacagctatgaccATCTCGAGCAGCTGAAgctccaatgtggtggaattcgtcg3′. **60S ribosomal protein**: 5′cgcatccgcgcaatgaAGACCATCCAGAGCAAtcagatcgtggacatacccgac3′. ***Bsep:*** 5′gtgtctcaggagccggtgttgTTCGACTGCAGCATTGccgacaacattcgctacggtgcc3′. ***Slc10a1:*** 5′ctgtcccggagggaacctctccaacgtgttcgcgctggcgcTCGACGGAGACATGAAcctcagcatcctcatgaccacgtg3′. ***Col2a1:*** 5′ttcacttactctgtgctggaggatgggTGCACTACGCACACCGgcgtgtggggcaagacggtgatcgagtacagg3′

***Hgf*** (using SYBR Green method, no probe was used): 5′cggcattgcttggaaggaaaaggggaaaattaccgcggccttgtgaacaaaacagccaccgac3′.***Hsp90:***

5′cgtgctgcacctgaaggaggaCCAATCTGAGTACCTGGAGgagaagcgcatcaaagacatcg3′. ***Met:*** 5′ctgcagacgcagaggttcaccACCAAGTCGGATGTGTGgtcgtttggcgttctgctg3′.

## Additional file

Additional file 1:
**List of genes and gene ontology categories for Fig. 1 and Fig. S1.** Hsp90 siRNA synchronized gall bladder degeneration during sea lamprey metamorphosis. (DOCX 4040 kb)

## Abbreviations

BA: Biliary atresia
ECM: Extracellular matrix
GO: Gene ontology
*hgf*: Hepatocyte growth factor
*hmgcr*: HMGCoA reductase
i.p.: Intraperitoneal
JV: Juvenile
L: Late larval stage
LP: Large parasite
M1-M7: Metamorphic stage 1-7
MSU: Michigan State University
OF: Ovulatory female
PSM: Pre-spermiating male
p-H3: Phosphohistone 3
POF: Pre-ovulatory female
RTQ-PCR: Real-time quantitative PCR
SM: Spermiating male
SP: Small parasite
T: Newly transformed juvenile
T_3_: Triiodothyronine
