# Supplementary material for: Hsp90 and hepatobiliary transformation during sea lamprey metamorphosis
Source: BMC Dev Biol. 2015 Dec 1;15:47. doi: 10.1186/s12861-015-0097-2 (PMC4667476; doi:10.1186/s12861-015-0097-2)
Supplement: Additional file 1: — List of genes and gene ontology categories for Fig. 1 and Fig. S1. Hsp90 siRNA synchronized gall bladder degeneration during sea lamprey metamorphosis. (DOCX 4040 kb) [file 12861_2015_97_MOESM1_ESM.docx]

**Supplementary Materials:**

Fig.1 Legend: **(A) X-axis**: 1.GO0048513 organ development, 2.GO0048856 anatomical structure development, 3.GO0048731 system development, 4.GO0007275 multicellular organismal development, 5.GO0006950 response to stress, 6.GO0065008 regulation of biological quality, 7.GO0023033 signaling pathway, 8.GO0007166 cell surface receptor linked signaling pathway, 9.GO0007010 cytoskeleton organization, 10.GO0009653 anatomical structure morphogenesis, 11.GO0000087 M phase of mitotic cell cycle, 12.GO0000280 nuclear division, 13.GO0007067 mitosis, 14.GO0048285 organelle fission, 15.GO0007049 cell cycle, 16.GO0000278 mitotic cell cycle, 17.GO0000279 M phase, 18.GO0022402 cell cycle process, 19.GO0022403 cell cycle phase, 20.GO0044093 positive regulation of molecular function, 21.GO0032879 regulation of localization, 22.GO0051049 regulation of transport, 23.GO0040012 regulation of locomotion, 24.GO0006928 cellular component movement, 25.GO0040011 locomotion, 26.GO0016477 cell migration, 27.GO0051674 localization of cell, 28.GO0048870 cell motility, 29.GO0008285 negative regulation of cell proliferation, 30.GO0006955 immune response, 31.GO0030029 actin filament-based process, 32.GO0000226 microtubule cytoskeleton organization, 33.GO0007017 microtubule-based process, 34.GO0007051 spindle organization, 35.GO0007052 mitotic spindle organization, 36.GO0006952 defense response, 37.GO0051707 response to other organism, 38.GO0009607 response to biotic stimulus, 39.GO0048015 phosphoinositide-mediated signaling, 40.GO0019932 second-messenger-mediated signaling, 41.GO0001775 cell activation, 42.GO0030031 cell projection assembly, 43.GO0030030 cell projection organization, 44.GO0048858 cell projection morphogenesis, 45.GO0006800 oxygen and reactive oxygen species metabolic process, 46.GO0031589 cell-substrate adhesion, 47.GO0007160 cell-matrix adhesion, 48.GO0051101 regulation of DNA binding, 49.GO0051090 regulation of transcription factor activity, 50.GO0090046 regulation of transcription regulator activity, 51.GO0001816 cytokine production, 52.GO0033157 regulation of intracellular protein transport, 53.GO0060341 regulation of cellular localization, 54.GO0051223 regulation of protein transport, 55.GO0032880 regulation of protein localization, 56.GO0070201 regulation of establishment of protein localization, 57.GO0051050 positive regulation of transport, 58.GO0051222 positive regulation of protein transport, 59.GO0050663 cytokine secretion, 60.GO0051047 positive regulation of secretion, 61.GO0050708 regulation of protein secretion, 62.GO0009123 nucleoside monophosphate metabolic process, 63.GO0009124 nucleoside monophosphate biosynthetic process, 64.GO0050953 sensory perception of light stimulus, 65.GO0007601 visual perception, 66.GO0030198 extracellular matrix organization, 67.GO0043062 extracellular structure organization, 68.GO0007059 chromosome segregation, 69.GO0000819 sister chromatid segregation, 70.GO0000070 mitotic sister chromatid segregation, 71.GO0030261 chromosome condensation, 72.GO0007076 mitotic chromosome condensation, 73.GO0071103 DNA conformation change, 74.GO0006323 DNA packaging, 75.GO0007586 digestion, 76.GO0002443 leukocyte mediated immunity, 77.GO0048729 tissue morphogenesis, 78.GO0055008 cardiac muscle tissue morphogenesis, 79.GO0060415 muscle tissue morphogenesis, 80.GO0007416 synapse assembly, 81.GO0048589 developmental growth, 82.GO0048638 regulation of developmental growth, 83.GO0000726 non-recombinational repair, 84.GO0090068 positive regulation of cell cycle process, 85.GO0006298 mismatch repair, 86.GO0015909 long-chain fatty acid transport, 87.GO0006691 leukotriene metabolic process, and 88.GO0043449 cellular alkene metabolic process. **Y-axis**:1.PLA2G1B, 2.DRD2, 3.THBS1, 4.TTN, 5.BBS2, 6.CHRNB2, 7.ANXA3, 8.ARRB2, 9.MSH6, 10.PTPRC, 11.NPM1, 12.NUSAP1, 13.SMC2, 14.NCAPD3, 15.NCAPH, 16.TARDBP, 17.BIRC5, 18.PBK, 19.KIF15, 20.KIF22, 21.CENPF, 22.CIT, 23.BRCA2, 24.NDC80, 25.SPAG5, 26.TUBG1, 27.STMN1, 28.SPC25, 29.PRC1, 30.KIF11, 31.KIF23, 32.UBE2C, 33.TPX2, 34.AURKA, 35.BUB1B, 36.CDH13, 37.NF2, 38.SERPINE2, 39.IGFBP5, 40.PDGFRA, 41.FN1, 42.CD2AP, 43.TRY4, 44.CUZD1, 45.ITGB1BP1, 46.ACE, 47.COL5A1, 48.ANGPTL3, 49.CEACAM1, 50.SYK, 51.MRE11A, 52.LCP1, 53.UNC13D, 54.CXCR4, 55.TNNC1, 56.ACTC1, 57.MYL3, 58.RPS27A|UBB, 59.MAPT, 60.TUBB3, 61.CYFIP1, 62.SEMA3A, 63.SEMA6A, 64.NOG, 65.SALL1, 66.CASC5, 67.BBS1, 68.TTLL3, 69.ABCA1, 70.TOP2A, 71.NLRC3, 72.HELLS, 73.RECQL4, 74.NCF2, 75.TUBB2C, 76.PAK1, 77.TLR6, 78.GRB10, 79.ITGAV, 80.ADCY2, 81.ADK, 82.AKAP12, 83.GABBR2, 84.GP1BA, 85.GP1BB, 86.SLC7A5, 87.FPR1, 88.OPRM1, 89.GRK5, 90.RGS3, 91.RGS20, 92.PTPRD, 93.PREX2, 94.LAMA1, 95.GRIA4, 96.GABBR1, 97.BAI3, 98.DOK1, 99.CAP1, 100.FBN2, 101.IGF2BP3, 102.TPPP3, 103.MAD2L1, 104.CCNA2, 105.DGKZ, 106.MAP2K6, 107.EGFL6, 108.GAS7, 109.RAD52, 110.DNM2, 111.POLA1, 112.ITGB3, 113.SOD2, 114.TYMS, 115.PCNA, 116.MCTP1, 117.PLCH1, 118.CFL1, 119.CHIT1, 120.IFI44, 121.ISG20, 122.MUC5AC, 123.COL4A6, 124.COL6A2, 125.SDR16C5, 126.GJA8, 127.PITPNA, 128.FBLN5, 129.LTC4S, 130.ALOX5, 131.ALOX5AP, 132.MYL7, 133.MYO1E, 134.PLEK2, 135.DOCK2, 136.FERMT2, 137.GSN, 138.PFN2, 139.PVRL1, 140.SEMA3C, 141.FTH1, 142.S100A11, 143.FABP3, 144.FABP6, 145.XRCC5, 146.ARNT, 147.SHANK1, 148.TMEM97, 149.SLC4A11, 150.SLC30A2, 151.SLC12A6, 152.RPS17, 153.RHAG, 154.PCSK2, 155.CUBN, 156.AGR2, 157.CHGA, 158.GNA12, 159.PHF17, 160.DNALI1, 161.SSBP1, 162.ABL2, 163.MAP2K3, 164.SLC15A1, 165.CTRL, 166.FABP2, 167.ATR, 168.LIMD1, 169.ZMYM6, 170.TNFRSF1A, 171.UMOD, 172.MSH3, 173.PMS2, 174.RAC2, 175.AOX1, 176.CAT, 177.PRDX2, 178.TNC, 179.RFC5, 180.RFC3, 181.PARP3, 182.NUDT1, 183.MBD4, 184.CCNO, 185.CYGB, 186.MGLL, 187.CSF3R, 188.GPR68, 189.HSP90AB1, 190.DNAJA1, 191.HSP90AA1, 192.ANXA2, 193.SYNGR3, 194.ABLIM1, 195.COL1A1, 196.DNM3, 197.CXCL1, 198.GPX3, 199.MPV17, 200.NEB, 201.COL12A1, 202.PLOD1, 203.KRT9, 204.PITX2, 205.FABP1, 206.NCL, 207.FABP7, 208.SPEG, 209.POU3F1, 210.SCN2A1, 211.TLL1, 212.STMN2, 213.SPARC, 214.PMP22, 215.PHYH, 216.MAB21L2, 217.LSAMP, 218.EXT1, 219.CDH11, 220.ATN1, 221.ADAM22, 222.AHNAK, 223.UPK1A, 224.SMARCA1, 225.LAMA2, 226.HCK, 227.FBN1, 228.CASP14, 229.CAPN3, 230.BTD, 231.ALOX12B, 232.ATP2A2, 233.NOTCH1, 234.GPR98, 235.ACVR2B, 236.PRDM16, 237.UTP11L, 238.HES1, 239.PTN, 240.CDKN2A, 241.TCF7L2, 242.BCL3, 243.CHIA, 244.TLR2, 245.NLRP3, 246.NOD2, 247.FLNA, and 248.RHOQ. **(B) X-axis**: 1.GO0006457 protein folding, 2.GO0055085 transmembrane transport, 3.GO0071496 cellular response to external stimulus, 4.GO0031668 cellular response to extracellular stimulus, 5.GO0031669 cellular response to nutrient levels, 6.GO0009119 ribonucleoside metabolic process, 7.GO0018196 peptidyl-asparagine modification, and 8.GO0018279 protein amino acid N-linked glycosylation via asparagines. **Y-axis**: 1.USF2, 2.USF1, 3.RPTOR, 4.HDAC6, 5.BMPR2, 6.ATG4C, 7.ATG16L1, 8.ATG3, 9.TUSC3, 10.STT3B, 11.STT3A, 12.RPN1, 13.RPN2, 14.TBCC, 15.SIL1, 16.SEP15, 17.RUVBL2, 18.PIGK, 19.PDIA6, 20.PDIA5, 21.NFYC, 22.LRPAP1, 23.HSPA1A, 24.ERO1LB, 25.DNAJC7, 26.DNAJA3, 27.AHSA1, 28.CALR, 29.UMPS, 30.GARS, 31.CTPS, 32.COASY, 33.ACLY, 34.CDA, 35.SLC23A2, 36.SLC23A1, 37.SLC19A2, 38.PEX5, 39.GRPEL1, 40.GIF, 41.GC, 42.ATP6V1H, 43.ATP2A1, and 44.ATP5O. **(C) X-axis**: 1.GO0000278 mitotic cell cycle, 2.GO0000279 M phase, 3.GO0022402 cell cycle process, 4.GO0022403 cell cycle phase, 5.GO0006260 DNA replication, 6.GO0009888 tissue development, 7.GO0007398 ectoderm development, 8.GO0008544 epidermis development, 9.GO0048015 phosphoinositide-mediated signalling, 10.GO0019932 second-messenger-mediated signalling, 11.GO0043449 cellular alkene metabolic process, 12.GO0006691 leukotriene metabolic process, 13.GO0033559 unsaturated fatty acid metabolic process, 14.GO0006690 eicosanoid metabolic process, 15.GO0007052 mitotic spindle organization, and 16.GO0007051 spindle organization. **Y-axis**: 1.POLA1, 2.TIPIN, 3.KRT7, 4.SLBP, 5.MRE11A, 6.RAD51, 7.HAUS2, 8.TUBG1, 9.RAD52, 10.MSH6, 11.RAD51L1, 12.CENPF, 13.SIRT2, 14.PFTK1, 15.DNM2, 16.DBF4, 17.BCAT1, 18.CDKN2A, 19.UBE2S, 20.SMC2, 21.PLK1, 22.PBK, 23.NUSAP1, 24.NCAPH, 25.L3MBTL, 26.KNTC1, 27.KIF22, 28.BIRC5, 29.CIT, 30.TPX2, 31.STMN1, 32.SPC25, 33.RAN, 34.PRC1, 35.KIF11, 36.KIF23, 37.NDC80, 38.UBE2C, 39.AURKA, 40.BUB1B, 41.PTGES3, 42.HPGD, 43.PLA2G10, 44.PLA2G1B, 45.PTGR1, 46.LTC4S, 47.ALOX5, 48.ALOX5AP, 49.TCFAP2A, 50.UPK1A, 51.TNNC1, 52.NOTCH1, 53.NOG, 54.MYL6, 55.MYL2, 56.IGFBP3, 57.FKBP1A, 58.DNAJA3, 59.BAMBI, 60.ACTC1, 61.ACVR2B, 62.RRM2, 63.KRT9, 64.KRT17, 65.KRT13, 66.EDAR, 67.CTGF, 68.COL5A2, 69.COL5A1, 70.COL1A2, 71.COL1A1, 72.BTD, 73.AQP3, 74.ANXA1, 75.ADAM9, 76.ALOX12B, 77.TYMS, 78.TOP2A, 79.RFC4, 80.FEN1, 81.PCNA, 82.SLC7A5, 83.NPY1R, 84.CDH13, 85.FPR1, 86.PLCH1, 87.DRD2, 88.HIST1H4A, 89.SHC1, 90.RRM1, 91.RFC5, 92.RFC3, 93.POLE2, 94.ORC4L, 95.NF2, 96.HMGB1, 97.GMNN, 98.ADSS, 99.ATR, 100.RPS27L, 101.MAD2L1, 102.CCNA2, 103.DGKZ, 104.PA2G4, 105.GAS7, and 106.MAP2K6. **(D) X-axis**: 1.GO0044281 small molecule metabolic process, 2.GO0003008 system process, 3.GO0042180 cellular ketone metabolic process, 4.GO0006082 organic acid metabolic process, 5.GO0019752 carboxylic acid metabolic process, 6.GO0043436 oxoacid metabolic process, 7.GO0006520 cellular amino acid metabolic process, 8.GO0009308 amine metabolic process, 9.GO0044106 cellular amine metabolic process, 10.GO0006519 cellular amino acid and derivative metabolic process, 11.GO0006066 alcohol metabolic process, 12.GO0061061 muscle structure development, 13.GO0050953 sensory perception of light stimulus, 14.GO0007601 visual perception, 15.GO0050954 sensory perception of mechanical stimulus, 16.GO0007605 sensory perception of sound, 17.GO0007600 sensory perception, 18.GO0050890 cognition, 19.GO0044272 sulfur compound biosynthetic process, 20.GO0007270 nerve-nerve synaptic transmission, 21.GO0015837 amine transport, 22.GO0015844 monoamine transport, 23.GO0015850 organic alcohol transport, 24.GO0051937 catecholamine transport, 25.GO0006725 cellular aromatic compound metabolic process, 26.GO0006575 cellular amino acid derivative metabolic process, 27.GO0006576 cellular biogenic amine metabolic process, 28.GO0044283 small molecule biosynthetic process, 29.GO0046483 heterocycle metabolic process, 30.GO0009124 nucleoside monophosphate biosynthetic process, and 31.GO0055086 nucleobase nucleoside and nucleotide metabolic process. **Y-axis**: 1.PTGS1, 2.QPRT, 3.PLA2G15, 4.IDH1, 5.ALDH8A1, 6.FASN, 7.FTCD, 8.MTHFD1L, 9.SDS, 10.CBS, 11.ADI1, 12.CTH, 13.HPD, 14.FN3K, 15.AADAT, 16.KMO, 17.NFS1, 18.MARS2, 19.GGT1, 20.GOT1, 21.IDO2, 22.ALDH6A1, 23.CAD, 24.PLA2G4A, 25.GAMT, 26.GATM, 27.TGFB2, 28.AMD1, 29.AGPAT6, 30.CHKB, 31.DRD2, 32.NF1, 33.GNAS, 34.GRM7, 35.RPIA, 36.DERA, 37.REXO2, 38.ENPP3, 39.NT5C3, 40.TSTA3, 41.NSUN2, 42.HELLS, 43.DNMT3A, 44.CXXC1, 45.AHCY, 46.AOF1, 47.ST3GAL6, 48.SLC37A4, 49.PKM2, 50.OSBPL1A, 51.LARGE, 52.FBP2, 53.DYRK2, 54.ALDH2, 55.CELA3B, 56.NDST1, 57.EXT1, 58.GALNT5, 59.SLC6A2, 60.SLC6A8, 61.NAT2, 62.SLC22A3, 63.TCF7L2, 64.ZFHX3, 65.SVIL, 66.SORT1, 67.SEMA4C, 68.MRAS, 69.MEF2C, 70.LAMA2, 71.MEF2A, 72.RASGRF1, 73.KIF1B, 74.USP46, 75.UNC13B, 76.TPP1, 77.TNNI1, 78.SYT1, 79.SLC12A6, 80.POU3F1, 81.PCLO, 82.NFAT5, 83.DES, 84.CHGA, 85.ACE, 86.CASQ2, 87.UTRN, 88.TAZ, 89.ACTC1, 90.CHRNA1, 91.PDXK, 92.ADK, 93.UMPS, 94.CAP2, 95.ADCY1, 96.ADCY2, 97.FECH, 98.PAICS, 99.XDH, 100.CHRNA4, 101.CRYM, 102.SDR16C5, 103.OAT, 104.RABGGTA, 105.ALDH7A1, 106.KCNMA1, 107.PCDH15, 108.COL2A1, 109.GPR98, 110.CHRNB2, and 111.DIAP1.

Fig. S1. *Hsp90* siRNA synchronized gall bladder degeneration during sea lamprey metamorphosis. Sea lamprey larvae at metamorphic stage 2 (M2) were treated with vehicle (3.3% lipofectamine, Life Technologies, Grand Island, NY, USA; i.p. injection) or 53.67µg/ml *hsp90* siRNA (Stealth RNAi duplex with sense sequence: 5’GCAGCAAAGUGGCGUAUUA3’, and antisense sequence: 5’UAAUACGCCACUUUGCUGC3’, Life Technologies; i.p. injection, 50µl/g body weight). Animals were euthanized with 0.02% MS222 (Sigma, St. Louis, MO, USA) after four months. Liver photographs were taken immediately after dissection.
